# Smoking Cessation after Cancer Diagnosis and Enhanced Therapy Response: Mechanisms and Significance

**DOI:** 10.3390/curroncol29120782

**Published:** 2022-12-17

**Authors:** Srikumar Chellappan

**Affiliations:** Department of Tumor Biology, H. Lee Moffitt Cancer Center and Research Institute, 12902 USF Magnolia Drive, Tampa, FL 33612, USA; chellappan@aol.com

**Keywords:** nicotine, smoking, survival, immunotherapy, drug resistance 5

## Abstract

The adverse effects of smoking on human health have been recognized for several decades, especially in the context of cancer. The ability of tobacco smoke components, including tobacco-specific carcinogens and additive compounds such as nicotine, to initiate or promote tumor growth have been described in hundreds of studies. These investigations have revealed the tumor-promoting activities of nicotine and other tobacco smoke components and have also recognized the ability of these agents to suppress the efficacy of cancer therapy; it is now clear that smoking can reduce the efficacy of most of the widely used therapeutic modalities, including immunotherapy, radiation therapy, and chemotherapy. Several studies examined if continued smoking after cancer diagnosis affected therapy response; it was found that while never smokers or non-smokers had the best response to therapy, those who quit smoking at the time of diagnosis had higher overall survival and reduced side-effects than those who continued to smoke. These studies also revealed the multiple mechanisms via which smoking enhances the growth and survival of tumors while suppressing therapy-induced cell death. In conclusion, smoking cessation during the course of cancer therapy markedly increases the chances of survival and the quality of life.

## 1. Introduction

Cancer places a huge burden on families and individuals across the world and is one of the leading causes of mortality worldwide [1]. Several behavioral and environmental factors are thought to contribute to the genesis of cancer, and it is accepted that enhanced public health awareness can contribute to reducing mortality, in addition to the development of novel therapeutic strategies. Recent reports suggest a marked reduction in the rate of cancer-related mortality, and this is attributed to changes in behaviors, public health awareness, and new therapies [2]. The most important change that contributed to the reduction in mortality is the reduced use of tobacco products, especially the smoking of cigarettes and similar products, such as bidis or cigars.

The US Surgeon General report in 1964 formally outlined the various adverse effects of smoking on human health for the first time, especially the correlation between smoking and cancer incidence [3]. Subsequent studies showed that smoking is associated with cancer of the lungs, oropharynx, pancreas, kidney, bladder and ureter, and stomach, as well as acute myeloid leukemia, etc. The Surgeon General 2014 report on smoking added liver cancer and colorectal cancer to the list [3]. Additional publications by the Surgeon General indicated an increased trend of smokeless tobacco use in children and young adults, which has the potential to lead to smoking in later years [4]. A comprehensive meta-analysis of available data that was published recently showed a direct correlation between smoking and sixteen cancer types; there was also an increased incidence of other diseases [5]. These findings highlight the increased need for smoking cessation to reduce the risk of developing cancer and several other serious maladies associated with smoking.

It has also become clear that smoking not only causes cancer but can also affect several facets of cancer biology. Thus, tobacco smoke and its components can initiate carcinogenesis, promote the progression and metastasis of cancer, and markedly reduce the efficacy of cancer therapy [6]. Increased smoking has been correlated with poor overall response to the most common therapeutic modalities used to combat cancer, including radiotherapy, chemotherapy, and immunotherapy. Most notably, it has become clear that smoking cessation, even at cancer diagnosis, has a marked positive impact on patients; this is due to the increase in the efficacy of treatment in the absence of tobacco smoke components and to the reduced side-effects [7]. This article provides an overview of the current understanding of how smoking affects the efficacy of cancer therapies, the underlying mechanisms, and how smoking cessation is critical while undergoing treatment for cancer. A literature search was conducted in the PubMed database on studies that describe the correlation between smoking and therapy response. Search terms included smoking and immunotherapy response; smoking and immune response; smoking and inflammation and cancer; smoking and radiotherapy; smoking and chemotherapy; smoking and chemotherapy resistance; smoking cessation and therapy response; nicotine and drug resistance; nicotine and apoptosis; smoking and cancer survival; smoking and mechanisms of resistance; nicotinic acetylcholine receptors and drug resistance. Studies with clinical trials that examine smoking status and therapy response were selected as primary sources of information, along with papers that describe the underlying molecular mechanisms.

## 2. Smoking and Immunotherapy Efficacy

Immunotherapy using immune checkpoint inhibitors is highly effective against lung cancers, including both small-cell and non-small-cell lung cancers [8]. Several smoking-related cancers, such as head and neck cancer, bladder cancer, etc., also respond to immunotherapy [9,10,11]. Not surprisingly, checkpoint inhibitors such as Pembrolizumab (Keytruda), Nivolumab (Opdivo), Ipilimumab (Yervoy), Duryalumab (Imfinzi), Cemiplimab (Libtayo), and Atezolizumab (Tecentriq) are widely used as first-line therapy against non-small-cell lung cancer (NSCLC) based on the programmed death-ligand 1 (PD-L1) status of the tumors [12,13]. Checkpoint inhibitors are known to be significantly more effective in tumors with higher mutational burden, including lung cancers and skin cancers such as melanoma [14,15]. The efficacy of immunotherapy in lung cancer patients based on their smoking status thus calls for detailed examination [16], since tobacco smoke is known to induce DNA damage and mutations in several oncogenes and tumor suppressor genes [17,18], resulting in higher mutational burden [19]; this has been validated in multiple tumor types, including squamous cell carcinomas of the lung, and head and neck [20,21]. It has been suggested that the prevalence of cancer-specific mutated peptides, which are generated as a result of somatic mutations caused by smoking, might increase the prevalence of neo-antigen-specific T cells, enhancing the response to checkpoint blockade [22]. While smoking and higher mutational burden might lead to a better response to immunotherapy [23], the inflammation and overall tumor promotion caused by smoking can be expected to negate the beneficial effects of any therapeutic modality [22]. At the same time, non-smokers generally have a lesser mutational burden, along with the absence of the tumor-promoting effects of tobacco smoke, which might affect the response to immunotherapy [24]. This conundrum has been addressed in several studies in the past few years.

A recent case–control matched analysis compared the relative efficacy of first-line chemotherapy and immunotherapy in smokers and non-smokers. This study, conducted on 962 NSCLC patients who had received first-line Pembrolizumab and 462 patients who had been treated with first-line platinum-based chemotherapy, found that never smokers had a significantly higher risk of disease progression when treated with Pembrolizumab [16]. There was no significant risk of disease progression in non-smokers treated with chemotherapy. This study further conducted random case–control matching on 424 patients from the Pembrolizumab and chemotherapy cohorts; it was found that the never smokers in the Pembrolizumab cohort had significantly shorter progression-free survival, whereas those in the chemotherapy cohort had significantly longer progression-free survival. Taken together, the authors concluded that smokers with NSCLC who were eligible for immunotherapy had better progression-free survival, especially in the earlier stages, while non-smokers benefited more from chemotherapy [16]. Similar studies conducted by other groups in different settings have led to the same conclusion—non-smokers might benefit more from chemotherapy and targeted therapy [25]. This was further supported by a metanalysis of published data. At the same time, the Keynote-024 study demonstrated an increased benefit from smoking cessation in patients undergoing immunotherapy [26]. These findings might appear to be counterintuitive, but perhaps, smokers in the earlier stages of lung cancer might respond better to immunotherapy than non-smokers.

The biochemical mechanisms underlying the increased efficacy of immunotherapy in smokers have been explored by several groups, and factors other than increased mutational burden have been found to be involved. It has been established that those patients with PD-L1 expression in more than 50% of cells are more responsive to frontline immune checkpoint inhibitors [27]. Multiple studies have found that nicotine and tobacco smoking in general induce the levels of PD-L1 in lung cancer cells [28]. A comparison of PD-L1 levels in smokers and non-smokers with K-Ras mutant NSCLC showed higher levels of PD-L1 in smokers, and this was associated with pack-years; such correlations were not observed in the levels of PD-L2 [27]. While high levels of PD-L1 neutralize the anti-tumor activity of T cells, this also renders the patient more sensitive to immune checkpoint inhibitor therapy. It should be noted, however, that exposure to tobacco smoke components induces inflammation as well as the expression of various proteins (such as YAP1) that are known to have immunosuppressive effects [29,30,31]. Despite these negative effects on tumor growth and immune response, clinical trials appear to vindicate that immunotherapy is an effective therapeutic strategy to combat NSCLC in smokers [32].

The effects of smoking on immune cells in the context of cancer have been elucidated in multiple studies [33]. It is known that smoking can affect the majority of immune cell types involved in adaptive and innate immunity; the increase in T helper cells, especially the Th17 subset, has been reported, along with the increase in several inflammatory interleukins. Similarly, CD8+ T cells are elevated in response to smoking, along with their functional responses. On the contrary, CD+ T cells, especially regulator T cells (Tregs), are downregulated in smokers, perhaps contributing to inflammation [22]. In conclusion, it is clear that smoking can generally increase inflammation and associated diseases, such as COPD (chronic obstructive pulmonary disease), and adversely affect the quality of life [34,35]. While smokers might benefit from immunotherapy, especially in the early stages of cancer, it is clear that smoking cessation subsequent to cancer diagnosis would not reduce the response to immunotherapy, but on the contrary, it would improve the quality of life [36].

## 3. Smoking and Radiotherapy

Several studies have demonstrated a clear correlation between smoking and the response to radiation or chemoradiation therapy in various cancers [37]. While the underlying molecular mechanisms have not been fully elucidated, the poor response to radiotherapy has been prevalent across several cancer types, indicating that it is a genuine correlation [38]. For example, head and neck cancer (H&N) patients are widely treated with radiation or chemoradiation, and many such tumors are strongly correlated with smoking [39,40]. A study conducted as early as 1993 categorically concluded that patients with head and neck cancer who had continued to smoke during radiation therapy had a significantly lower rate of response and lower overall survival than those who did not smoke [41]. The study was conducted on 135 patients undergoing radiation therapy, of which 53 were smokers and 62 were non-smokers during therapy. Similar results were obtained in several subsequent studies; for example, a study using a questionnaire was conducted on 115 H&N cancer patients who were treated with radiation therapy with or without 5-fluorouracil. After adjusting for various variables, it was found that patients who had continued to smoke during radiation therapy had lower complete response rates; this study also showed that the response in non-smoking patients increased depending on the duration between quitting and treatment. Those who had quit at least one year before the commencement of treatment had better response than those who had quit less than 12 weeks prior to diagnosis [41]. Other studies on H&N patients showed that patients who had been active smokers during radiation therapy had markedly lower overall survival and disease-free survival than subjects who had quit before the commencement of radiation therapy [42]. These studies also suggested that the longer one has abstained from smoking, the better the response one obtains from radiotherapy is. A summary of these findings is provided in Table 1.

A trial was conducted in 2012 based on reports that head and neck patients having higher levels of normoxia markers responded well to radiotherapy, while those with hypoxic markers, including low hemoglobin, responded poorly [43]. Making the assumption that increased hypoxia was correlated with smoking status, a prospective study was conducted on two hundred and thirty-two patients with head and neck squamous cell carcinoma. Out of these, 108 were heavy smokers, while the remaining were moderate smokers or quitters. The study showed that there was a linear correlation between carboxyhemoglobulin levels and increased smoking status. A univariate analysis showed that heavy smokers had reduced disease-specific and overall survival compared with non-smokers. There was reduced loco-regional control in the patients who were heavy smokers. The study concluded that there was a significant negative impact of smoking during radiotherapy for H&N cancer and that the risk increased with each additional pack-year of smoking [43]. In one of the more recent studies, smokers with head and neck squamous cell carcinomas who were undergoing chemoradiation were prospectively enrolled in a smoking cessation trial. It was found that 65% of the subjects discontinued smoking throughout the therapy; these subjects had significantly lower probability of acute toxicity, lower probability of gastrostomy or tracheostomy, and greater probability of progression-free survival. Overall, smoking cessation during chemoradiation therapy significantly reduced tumor progression risk in these patients [42]. Other studies have resulted in similar results and conclusions. An analysis of the effect of smoking and human papilloma virus (HPV) on the overall survival of oropharyngeal squamous cell carcinoma patients undergoing chemoradiation found that out of 120 patients, 71% had HPV-positive tumors and that patients who were current smokers and were HPV-positive had significantly worse overall survival [44]; HPV-negative patients who were smokers also had markedly poor overall survival compared with former or never smokers. After adjustments for age, gender, and tumor stage, HPV+ current smokers showed an almost four-fold increase in mortality compared with HPV- never smokers. In all the cohorts, smokers had significantly higher mortality rates than non-smokers [44]. In a broadly similar analysis on 237 non-small-cell lung cancer patients, two-year overall survival was assessed in patients from the initiation of chemoradiation. The study concluded that early-stage patients who were non-smokers had a significantly higher overall survival rate (56% survival rate and median survival of 27.9 months) than smokers (41% survival rate and median survival of 27.9 months). Surprisingly, there was no significant difference in survival among Stage III patients; the investigators concluded that smoking reduced the response to chemoradiation in early-stage patients [45].

The role of nicotinic acetylcholine receptors was evaluated in the context of resistance to radiation therapy, in an in-depth molecular analysis. Lin et al. (2019) reported that the nicotinic acetylcholine receptor subunit, α5, contributes to radiation resistance in oral squamous cell carcinomas [46]. They found that the knockdown of the α5 receptor enhanced radiation sensitivity to OSCC cells, but the addition of nicotine reduced radiosensitivity. The authors went on to demonstrate that the α5 nAChR subunit induced E2F transcription factor-mediated proliferative and survival pathways to confer resistance to radiation and proposed that the levels of α5 can potentially be used to predict radiotherapy response in OSCC patients [46]. Taken together, it is clear that exposure to tobacco smoke or its components, such as nicotine, could enhance the resistance to radiotherapy and that smoking cessation could confer sensitivity to radiation [47].

## 4. Smoking and Chemotherapy

Adverse effects of smoking on cancer chemotherapy have been recognized for decades. One of the earliest studies, published by John Minna, Paul Bunn, and colleagues in 1980 [48], stratified 112 small-cell lung cancer patients into three groups: those who had quit smoking prior to diagnosis, those who stopped at diagnosis, and those who continued to smoke. It was found that those who had quit smoking prior to diagnosis had the best survival among all patients, followed by those who quit smoking at diagnosis; those who continued to smoke had the lowest survival. The study concluded that quitting smoking even at the time of diagnosis had a notably beneficial effect on survival. It is notable that survivors of tobacco-related cancers show higher rates of smoking prevalence than survivors of non-tobacco-related cancers or those without a history of cancer [49]. A study published in 2006 analyzed the data from 1370 patients with AJCC Stage III or IV NSCLC who were treated with chemotherapy or chemoradiation; their results clearly showed improved overall survival rates among never-smokers who underwent chemotherapy [50].

Earlier studies also showed a correlation between the amount of lifetime smoking and therapy response. A retrospective study in Brazil [51] conducted on 283 lung cancer patients who received at least two cycles of platinum-based chemotherapy found that 33% of the patients that responded to chemotherapy had a smoking history of 38.7 ± 27.1 pack-years, but those who did not respond had smoked 67.8 ± 35.1 pack-years. The authors concluded that patients with a smoking history of more than 40 pack-years had a markedly worse response to chemotherapy than those who had smoked less over their lifespan.

Similar high prevalence rates of smoking in cancer survivors have been reported, calling for more robust educational efforts in determining the deleterious effects of smoking on survivorship and quality of life. A study conducted on 1118 cancer survivors surveyed their awareness of the harms of continued smoking during therapy; surprisingly, a large proportion of the subjects were unaware that continued smoking leads to poor therapy response, enhanced surgical complications, and decreased overall quality of life [52]. Current smokers were more unaware of these harms; the study also reported that awareness that continued smoking contributes to poor therapy outcome and enhanced side-effects led to smoking cessation in these patients [52]. Broadly similar results were reported earlier in NSCLC patients, where it was found that patients who had quit smoking after diagnosis had a better performance status that those who had continued to smoke [53]. This trend was found at 6 months and 12 months of follow-up and was evident irrespective of the patient characteristics. A different study also showed reduced quality of life in persistent smokers who were lung cancer survivors compared with non-smokers, as measured using a lung cancer symptom scale [54].

In addition, studies have been conducted on how smoking affects treatment-related symptom burden. A study in 2011 examined how smoking affected the side-effects of cancer treatment on 947 patients who were scheduled to undergo chemotherapy or radiation [7]. The effect of smoking status on common measures of side-effects were assessed during the course of treatment or during a 6-month follow-up. It was found that smokers had markedly high side-effects during treatment compared with non-smokers; this trend continued even at the 6-month follow-up. Interestingly, the investigators found that the subjects who had quit smoking prior to the commencement of therapy had lower symptom levels, which were comparable to those of non-smokers [7]. These findings further highlight the importance of not smoking during cancer therapy.

A more recent study on smoking and therapy efficacy was conducted on head and neck squamous cell carcinoma (HNSCC) patients who had quit smoking after cancer diagnosis, prior to the commencement of therapy [55]. The study on 137 patients reported that those who had quit smoking after HNSCC diagnosis were 3.7-fold more likely to respond to first-line therapy than the patients who continued to smoke and had higher chances of disease-free survival. Those who had not smoked during treatment had a markedly lower probability of mortality from all causes than smokers. These studies further highlight the importance of quitting before commencing cancer therapy and during the course of treatment.

Non-muscle-invasive bladder cancer patients who had quit smoking after diagnosis and prior to the commencement of therapy had a lower probability of tumor recurrence [56]. This was in agreement with other studies on different smoking-related cancer types that established a high correlation between smoking and tumor recurrence, compared with never smokers or those who quit smoking [57,58]. A different study conducted on the effect of smoking on the response of patients suffering from urothelial carcinoma of the bladder to neoadjuvant chemotherapy showed similar results [59]. A comparison of clinicopathological variables in 167 patients showed that former and current smoking statuses were strongly correlated with worse Eastern Co-operative Oncology Group (ECOG) performance status and decreased response to neoadjuvant therapy; current smokers had a markedly higher likelihood of no pathological response to neoadjuvant therapy. The authors also concluded that response to neoadjuvant chemotherapy was a strong indicator of survival; in addition, smokers might be better candidates for immunotherapy, as in the case of lung cancer. Additional studies also confirmed these observations on muscle-invasive bladder cancer [56]. A retrospective study of 201 patients indicated a significant association between smoking status and poor or no response to neoadjuvant therapy; further, current smoking status was correlated with increased probability of tumor recurrence [60].

Pancreatic cancer is one of the deadliest cancers, with short overall survival and low response to current therapies. Smoking is highly correlated with pancreatic cancer, and studies have shown that smokers have notably lower survival rates than non-smokers or those who quit after diagnosis. The reduction in survival is correlated with the increase in pack-years of smoking [61]. Several studies have indeed documented a correlation between smoking and the low overall survival of pancreatic cancer patients. It has also been demonstrated that one reason for the reduced survival is increased inflammation, which correlates with smoking and pancreatitis [62]. It is clear that different cancers that develop as a result of smoking have common as well as distinct mechanisms that contribute to poor therapy response and poor survival.

Smoking has been correlated with higher levels of inflammation in cancer patients and survivors [34]. One study on breast cancer survivors examined a novel aspect; they measured the inflammatory response in breast cancer survivors who were prior smokers or who were non-smokers. The study on patients who had completed their treatment at least three months prior [63] found that exposure to standardized laboratory stressors involving speech and mental arithmetic induced markedly higher levels of interleukin-6 (IL-6) in former smokers than in never smokers. The authors suggested that the increased inflammatory response to stress could have contributed to the persistent inflammation observed in smokers and former smokers, even years after quitting smoking or after cancer therapy.

## 5. Mechanisms by Which Smoking Affects Therapy Response

Tobacco smoke contains several carcinogens, such as tobacco-specific nitrosamines, poly-aryl hydrocarbons such as benzo[a]pyrene, aldehydes, benzene, aromatic amines, etc. [64,65]. While many of them are active mutagens that form adducts with DNA and alter vital tumor suppressor genes or oncogenes promoting tumor growth, other components of tobacco smoke can modulate the signaling events in cells to enhance cell proliferation, suppress apoptosis, and confer a survival advantage to the exposed cells. Several of the tobacco smoke components can induce abnormal DNA methylation or other epigenetic events, contributing to oncogenesis [66].

Smokers metabolize chemotherapy drugs faster than non-smokers [67]. When neutropenia was taken as a marker of toxicity mediated by chemotherapy drugs, it was found that non-smokers who were receiving gemcitabine treatment for various cancers had higher neutropenia than smokers. While lower neutropenia indicates a higher number of neutrophils and is more desirable under normal conditions, higher neutropenia shows a reduction in the levels of gemcitabine in the blood and lower efficacy of chemotherapy; this was observed in an analysis that spanned across multiple tumor types [68].

On the other hand, nicotine, which is the major addictive component of tobacco smoke, is a prime example of how a tobacco smoke component can promote tumor growth and metastasis without altering the genetic makeup of cells [69,70]. Nicotine by itself is not a carcinogen at the levels found in the bloodstream of heavy smokers, but high doses of nicotine have been reported to be carcinogenic, though via unknown mechanisms [71,72]. Two potent tobacco-specific carcinogens, nicotine-derived nitrosamine ketone (NNK) and N-nitrosonornicotine (NNN), are generated by nicotine during the curing of tobacco or during smoking [73]. The possibility exists that the high dose of nicotine used in those studies also gave rise to trace amounts of NNK or NNN, which might have contributed to the observed oncogenesis. Many of the tobacco-specific nitrosamines require metabolic activation, mediated by cytochrome P450 enzymes, which are in turn induced by exposure to tobacco smoke [67]. These enzymes are also involved in the detoxification of chemotherapy drugs, which effectively reduces their efficacy. The impact of smoking on the effectiveness of lung cancer therapy has been discussed in detail few years ago [74].

Nicotine by itself acts on cells through nicotinic acetylcholine receptors (nAChRs), which are expressed predominantly in neurons and neuro-muscular junctions [75,76,77]. These pentameric receptors are also expressed on a variety of epithelial and endothelial cells; it has been established that signaling through specific nAChR subunits facilitates the nicotine-mediated induction of cell proliferation and suppression of apoptosis. Interestingly, it was reported, several years ago, that alterations in specific nAChR subunits, including α3, α4, α5, β2, and β4, contribute to smoking addition and lung cancer [78,79,80]; these findings were obtained in genome-wide association studies that found single-nucleotide polymorphisms in gene clusters that code for the genes for these nAChR subunits. In a similar vein, α7 and certain other subunits are directly involved in promoting cell proliferation [81,82], while α3 β2 subunits contribute to the suppression of apoptosis [83]. In addition, nicotine has also been reported to promote the epithelial–mesenchymal transition, angiogenesis, stemness, and metastasis of various cancers through the mediation of nAChRs [70,84]. It is clear that nAChRs play a major role in facilitating several deleterious effects of smoking, especially in cancer patients.

Based on the roles of nAChRs in promoting tumor growth, metastasis, angiogenesis, and drug resistance, it has been suggested that they may be potential targets for therapy [85,86]. Several antagonists are available to specific nAChR subunits, including neuronal and muscle-type nAChRs. Unfortunately, most of these antagonists are highly toxic to humans; for example, bungaratoxin and cobratoxin, which are present in snake venom, are selective antagonists of the α7 subunit [87]. Similarly, hexamethonium bromide and α-conotoxin are other agents that can have deleterious effects on the body, thus eliminating their use as anti-cancer agents. Two FDA-approved nAChR antagonists are widely used in smoking cessation. Varenicline is an antagonist of α4β2 subunits, while bupropion affects α3/β2 and other subunit combinations; these agents have not demonstrated significant anti-cancer or anti-proliferative properties so far, probably since the subunits they target are not strongly functional in non-neuronal cells.

Several molecular mechanisms have been proposed for how smoking and nicotine in particular confer resistance to therapy. Earlier studies have described several molecular mechanisms that are driven by nAChR activation, especially certain specific subunits. While several different subunits have been demonstrated to be involved in resistance to therapy, especially α2/β3, α7, α5, etc., it is clear that the survival signals mediated by these subunits negate the effects of chemotherapy. For example, in cell culture models of NSCLC, it was found that pre-treatment with 1 μM nicotine efficiently suppressed apoptosis induced by several first-line chemotherapy agents, including cisplatin, gemcitabine, and taxanes [83]. This involved the induction of the anti-apoptotic X-linked inhibitor of apoptosis protein (XIAP) via protein stabilization and the induction of survivin at the transcriptional level. The induction of survivin by nicotine was also reported in oral cancer cells, where cisplatin-induced apoptosis was suppressed. Supporting these findings, in vivo mouse xenograft models showed that the administration of nicotine promoted the growth and metastasis of pancreatic cancers and also suppressed their response to gemcitabine.

In a similar vein, exposure to nicotine has been shown to activate the extracellular signal-regulated kinase (ERK) and protein kinase B (Akt kinase) pathways to promote the survival of colorectal cancer (CRC) cell lines [88,89]. NNK was found to upregulate the Akt pathway and nuclear factor kappa B (NFκB)-dependent signaling to promote the survival of small-cell lung cancer (SCLC) cells [89]. The upregulation of sirtuins, which is one of the multiple epigenetic mechanisms activated by nAChRs, is another proposed mechanism [90]. The activation of β-adrenergic receptors and downstream signaling events are also known to contribute to the tumor-promoting functions of nicotine [91].

The tumor-promoting activities of nicotine involve the inactivation of tumor suppressors such as retinoblastoma tumor suppressor protein (Rb) and p53; in addition, tobacco smoke components can induce oncogenic proteins such as c-Myc and K-Ras [92]. Nicotine has also been reported to induce cell proliferation by enhancing the levels of cyclin D and E, and their associated kinase activities; this leads to increased phosphorylation and inactivation of retinoblastoma tumor suppressor protein, Rb, resulting in enhanced proliferation of lung cancer cells. This involves the activation of Src through the mediation of β-arrestin 1. In addition to activating Src, β-arrestin 1 was found to translocate to the nucleus, where it physically interacted with the E2F1 transcription factor to induce the expression of various genes involved in cell proliferation, epithelial–mesenchymal transition (EMT), and metastasis [93]. Further, multiple matrix-metalloproteinases were found to be transcriptionally induced by nicotine through the mediation of E2F transcription factors [84]; thus, nicotine could promote cell survival even in the presence of chemotherapy drugs, facilitate cell proliferation, and promote tumor progression and the metastasis of cancer cells.

Cancer stem cells, often referred to as tumor-initiating cells, have gained attention in the past decade as mediators of drug resistance [94,95]. Cancer stem cells express higher levels of survival proteins; embryonic stem cell transcription factors such as Oct4, Sox2, and nanog; and drug exporters such as ABCG2. Studies by several groups, including ours, found that exposure to nicotine enhanced the self-renewal of stem-like cells from NSCLC cell lines [96]. This involved the induction of embryonic stem cell transcription factors such as Sox2, as well as oncogenic transcriptional co-activator YAP1. Interestingly, similar induction of stemness and self-renewal was observed when stem-like cells were treated with the contents of e-cigarettes [97]. Other studies have also found that nicotine induces stemness; for example, it was reported that the exposure of breast cancer cells to nicotine enhanced the levels of stem-like side-population cells and conferred resistance to chemotherapy drugs [98]. This involved the mediation of α9 nAChR and the induction of STAT3 and galectin 3. It thus appears that the induction of stemness is one of the mechanisms via which nicotine confers resistance to chemotherapy drugs.

As mentioned above, smoking affects the levels of chemotherapy agents in the system by altering their metabolism. A review article published few years ago provides precise and detailed information on how specific chemotherapy agents and targeted therapies are affected by smoking [67]. As outlined in that review article, components of tobacco smoke induce cytochrome P450 family proteins, such as CYP1A1 and CYP1A2 enzymes, which metabolize drugs such as erlotinib, reducing their effective levels. Further, enhanced levels of CYP1 enzymes and glucouronyl transferases significantly alter the pharmacokinetics and pharmacodynamics of several drugs, reducing their efficacy. The drugs altered by metabolic events include, but are not limited to, erlotinib, gemcitabine, taxanes, irinotecan, platinum compounds, etc. Furthermore, the CYP2A6 protein is known to metabolize nicotine itself and affect the incidence of COPD [99]. Overall, it is clear that the metabolic events mediated by components of tobacco smoke and the receptor-mediated events mediated by nAChRs confer significant survival advantage to cancer cells, markedly reducing the efficacy of systemic chemotherapy [67].

E-cigarettes, or electronic nicotine dispersal systems, have been touted as healthier alternatives to cigarettes. The reasoning behind this line of thinking is that E-cigarettes contain nicotine along with stabilizing and flavoring agents and thus lack the classic tobacco carcinogens [100]. E-cigarettes have been recommended as agents that can facilitate smoking cessation and might indeed have such benefits [101]. At the same time, given the presence of nicotine and the unknown effects of the flavoring and stabilizing agents, it can be imagined that prolonged use of E-cigarettes might have tumor-promoting activities comparable to those of nicotine and perhaps have deleterious effects on cancer therapy. The literature is scarce regarding how E-cigarettes affect the efficacy of chemotherapy, immunotherapy, or radiation, but it can be imagined that such information will be available in the coming years. While E-cigarettes are the most widely used nicotine dispersal system in use, other vaping devices, such as hookahs, might have similar effects.

Several pharmaceutical agents that function by modulating various nAChR subunits have been approved by the FDA for smoking cessation; these include drugs such as varenicline, cytisine, and bupropion, which do not have any demonstrated adverse effects on therapy response. These agents have different properties and are generally safe for most smokers. In addition, behavioral therapies have been successfully used; these approaches totally avoid the use of nicotine or nicotine replacement as a strategy to facilitate cessation. From the known adverse effects of nicotine and other components in nicotine replacement therapies, it is more desirable to use one of the pharmacological agents or behavioral therapy.

## 6. Conclusions

It is abundantly clear that smoking adversely affects the efficacy of anti-cancer therapies. Various components of tobacco smoke, including tobacco-specific carcinogens and nicotine, promote various survival signaling pathways in cancer cells and in the tumor microenvironment; further, they can alter the metabolism and degradation of drugs, reducing their effective concentrations and impacting their therapeutic benefit. In addition, the pro-inflammatory effects of tobacco smoke and nicotine alter the immune response and enhance the side-effects of therapy. These effects of tobacco smoke components, along with their negative effects on the overall physiology, result in poor survival outcomes.

Continued smoking after cancer diagnosis has been strongly correlated with poor overall outcome, and smoking cessation after diagnosis improves response to therapy. These facts strongly support increased educational efforts to promote smoking cessation after cancer diagnosis. E-cigarettes and similar nicotine supplements might have adverse tumor-promoting effects over prolonged use, and ideally, using these agents for smoking cessation should be limited to the shortest duration possible. It can be imagined that increased education on the benefits of quitting smoking and the availability of resources to promote smoking cessation will continue to reduce the incidence of cancer and lead to further reductions in cancer-related mortality.

## Figures and Tables

**Table 1:** Summary of the major findings on smoking status and therapy response in cancer patients.

Tumor Type	Smokers	Non-/Never Smokers	Therapy	Best Responders	Citation
NSCLC	864	98	Immunotherapy	Smokers	16
NSCLC	378	48	Chemotherapy	Non-smokers	16
H&N	53	62	Radiation	Non-smokers	41
H&N	63		Chemoradiation	Quitters (prior to therapy)	42
H&N	220	12	Radiotherapy	Non-smokers	43
H&N	94	26	Chemoradiation	Non-smokers	44
NSCLC	92	145	Radiation	Non-smokers	45
NSCLC	57	55	Chemotherapy	Non-smokers	48
NSCLC	1152	167	Chemoradiation/ Chemotherapy	Never smokers	50
NSCLC	255	30	Chemotherapy	Light smokers compared with heavy smokers	51
H&N	79	55	Chemoradiation	Quitters (compared with continued smokers)	
UCB	119	48	Chemotherapy	Non-smokers	55
MIBC	143	58	Chemotherapy	Non- or never smokers	60

NSCLC, non-small-cell lung cancer; H&N, head and neck cancer; UCB, urothelial carcinoma of the bladder; MIBC, muscle-invasive bladder cancer.
